# Morphological and physiological species-dependent characteristics of the rodent Grueneberg ganglion

**DOI:** 10.3389/fnana.2014.00087

**Published:** 2014-08-27

**Authors:** Julien Brechbühl, Magali Klaey, Fabian Moine, Esther Bovay, Nicolas Hurni, Monique Nenniger-Tosato, Marie-Christine Broillet

**Affiliations:** Department of Pharmacology and Toxicology, Faculty of Biology and MedicineUniversity of Lausanne, Switzerland

**Keywords:** olfactory subsystem, Grueneberg ganglion, rodent, electron microscopy, calcium imaging, chemo-sensitivity, thermo-sensitivity, alarm pheromone

## Abstract

In the mouse, the Grueneberg ganglion (GG) is an olfactory subsystem implicated both in chemo- and thermo-sensing. It is specifically involved in the recognition of volatile danger cues such as alarm pheromones and structurally-related predator scents. No evidence for these GG sensory functions has been reported yet in other rodent species. In this study, we used a combination of histological and physiological techniques to verify the presence of a GG and investigate its function in the rat, hamster, and gerbil comparing with the mouse. By scanning electron microscopy (SEM) and transmitted electron microscopy (TEM), we found isolated or groups of large GG cells of different shapes that in spite of their gross anatomical similarities, display important structural differences between species. We performed a comparative and morphological study focusing on the conserved olfactory features of these cells. We found fine ciliary processes, mostly wrapped in ensheating glial cells, in variable number of clusters deeply invaginated in the neuronal soma. Interestingly, the glial wrapping, the amount of microtubules and their distribution in the ciliary processes were different between rodents. Using immunohistochemistry, we were able to detect the expression of known GG proteins, such as the membrane guanylyl cyclase G and the cyclic nucleotide-gated channel A3. Both the expression and the subcellular localization of these signaling proteins were found to be species-dependent. Calcium imaging experiments on acute tissue slice preparations from rodent GG demonstrated that the chemo- and thermo-evoked neuronal responses were different between species. Thus, GG neurons from mice and rats displayed both chemo- and thermo-sensing, while hamsters and gerbils showed profound differences in their sensitivities. We suggest that the integrative comparison between the structural morphologies, the sensory properties, and the ethological contexts supports species-dependent GG features prompted by the environmental pressure.

## Introduction

In the nasal cavities of the mouse, close to the opening of the nares, a ganglion structure of unknown function was found in 1973 (Grüneberg, 1973). It was described as a heterogenic and compact structure situated between large blood vessels (BV). Histological investigations performed at the time allowed the identification of similar ganglion structures in other mammalian species (Grüneberg, 1973; Tachibana et al., 1990). It was rediscovered in 2005 (Fuss et al., 2005; Koos and Fraser, 2005; Fleischer et al., 2006a; Roppolo et al., 2006; Storan and Key, 2006) during inspection of whole-mount specimens from a particular gene-targeted OMP-GFP (olfactory marker protein-green fluorescent protein) mouse in which all mature olfactory neurons express GFP as a histological reporter under the control of the OMP promoter (Margolis, 1972; Mombaerts et al., 1996; Potter et al., 2001). This structure was further referred to as the Grüneberg ganglion or Grueneberg ganglion (GG) according to its first description (Grüneberg, 1973). The GG of OMP-GFP mice was found to start developing around embryonic day 16 and to be composed of two different cell types, neurons and glial cells. It appeared to be complete at birth and to persist throughout the mouse life (Fuss et al., 2005; Koos and Fraser, 2005; Fleischer et al., 2006a; Roppolo et al., 2006; Storan and Key, 2006; Brechbühl et al., 2008; Liu et al., 2009). Its localization in a water permeant epithelium that lacks direct contact with the nasal cavity and its general morphological aspect were further compared with the amphid neurons of the nematode *Caenorhabditis elegans* (*C. elegans*) (Brechbühl et al., 2008, 2013a) which respond both to chemo- and thermo-stimuli. The olfactory function of the GG was supported in the mouse by the expression of putative olfactory receptors (Fleischer et al., 2006b, 2007); the presence of numerous cilia (Brechbühl et al., 2008; Liu et al., 2009) and the direct connections to the necklace complex of the olfactory bulb of the brain (Koos and Fraser, 2005; Roppolo et al., 2006; Matsuo et al., 2012). Recently, we found the mouse GG to be involved in the recognition of volatile danger cues such as mouse alarm pheromones and other structurally-related molecules found in predator scents (Brechbühl et al., 2008, 2013b). Different technical approaches demonstrated that both chemo- and thermo-stimuli could initiate neuronal responses in the mouse GG (Brechbühl et al., 2008, 2013a; Mamasuew et al., 2008, 2011a; Schmid et al., 2010) partially using cyclic guanosine monophosphate (cGMP)-dependent pathways (Fleischer et al., 2009; Liu et al., 2009; Mamasuew et al., 2010, 2011b; Schmid et al., 2010; Brechbühl et al., 2013a; Hanke et al., 2013; Stebe et al., 2013).

To our knowledge no evidence for the multisensory functions observed in the mouse GG has been reported yet in other rodent species. We here investigated and confirmed by ultrastructural and immunohistochemical approaches the presence of a GG in the rat, hamster, and gerbil. Their olfactory features were compared with those observed in the mouse. We found that both the morphology and the physiological responses, measured by calcium imaging experiments, were different between the investigated rodent species. Thus, chemo- and thermo-evoked neuronal responses were correlated with the structural morphology and with the signaling proteins expressed. Similar chemo- and thermo-responses were observed in mouse and rat GG neurons, while in the hamster and gerbil profound sensitivity differences existed. We thus propose that the environmental and evolutionary pressure could explain the interspecies sensitivities displayed by the rodential Grueneberg ganglia.

## Materials and methods

### Animals and tissue preparation

Adults and pups C57BL/6J mice (*Mus musculus*), Wistar rats (*Rattus norvegicus*), Golden hamsters (*Mesocricetus auratus*), and Mongolian gerbils (*Meriones unguiculatus*) were used for experimental investigations and were ordered from Janvier Labs. Postnatal day is indicated as (P). Animals were euthanized by CO_2_ or cervical dislocation. Animal care was in accordance with the Swiss legislation and the veterinary authorities. Nasal cavities were prepared in ice-cold artificial cerebrospinal fluid (ACSF; 118 mM NaCl, 25 mM NaHCO_3_, 10 mM D-glucose, 2 mM KCl, 2 mM MgCl_2_, 1.2 mM NaH_2_PO_4_, and 2 mM CaCl_2_, pH 7.4) saturated with oxycarbon gas [95% O_2_: 5% CO_2_; (vol/vol)] under a dissecting microscope set-up (M165 FC; Leica).

### Scanning and transmitted electron microscopy observations

For scanning electron microscopy (SEM) and transmitted electron microscopy (TEM) procedures, coronal sections from GG regions were prepared according to Brechbühl et al. (2008). Briefly, animals were anesthetized with intraperitoneal injections of sodium pentobarbital. After fixation steps with 2% paraformaldehyde (PAF 2%; #158127, Sigma), 2.5% glutaraldehyde (Glut 2.5%; #16220, Electron Microscopy Sciences) in phosphate buffered saline (PBS; 138 mM NaCl, 2.7 mM KCl, 1.76 mM KH_2_PO_4_, and 10.0 mM Na_2_HPO_4_, pH 7.4), animal heads were delicately dissected and placed overnight in the same fixative solution at 4°C. They were then briefly rinsed in PBS, included in 5% agar (#A7002, Sigma) and fixed vertically with Roti® coll 1 cyanacrylat glue (#0258.1, Carl Roth) onto the holder of the vibroslicer (VT1200 S, Leica). 80–200 μm coronal sections were obtained and collected in 0.1 M cacodylate buffer (#20840, Sigma). These slices could be then used for SEM or TEM preparations. For SEM processing, selected sections were fixed in 0.2 M cacodylate buffer containing Glut 2.0% overnight at 4°C. After washing phases, samples were dehydrated at room temperature (RT; corresponded to 23–25°C) using increasing alcohol concentrations and dried by the critical point method. Finally, they were mounted on aluminum stubs, coated with platinum (12 nm) and examined with a scanning electron microscope (JSM-6300F, JEOL) operated at 5 to 15 kV accelerating voltage. For TEM processing, selected sections were placed in 1% osmium tetroxide in 0.1 M cacodylate buffer for 60 min at RT and then washed with distilled water. They were then transferred for 30 min of staining in 1% uranyl acetate and washed with distilled water. Sections were dehydrated in graded alcohol series and placed in propylene oxide. Embedding procedures were done in EPON®-DMP30 resin further replaced by fresh Durcapan® on glass slides and placed in an oven for 48 h at 65°C. 50–70 nm ultra-thin sections were prepared and membrane contrast was obtained by lead-citrate. Observations were made on a TEM microscope (CM 100 EDX, Philips).

### Immunohistochemistry

The floating immunohistochemistry procedure used was from Brechbühl et al. (2013a). Briefly, the tips of noses were transferred and dissected in PBS before being fixed in PAF 4% at 4°C for 3 h. Fixed tissue preparations were embedded in 5% agar (#A7002, Sigma) and 60-120 μm coronal sections were cut and collected in PBS. Slices were then blocked overnight at 4°C in a PBS solution containing 10% normal goat serum (NGS; #UP379030, Interchim) and 0.5% Triton® X-100 (#93420, Sigma). Primary antibodies were applied to the slices for 16 h at RT in NGS 5%, Triton® X-100 0.25%. Slices were washed in NGS 2% before incubation in the dark with the secondary antibody in NGS 2% for 1 h at RT. Finally, slices were washed and mounted in Vectashield® mounting medium (#H-1200, Vector Labs) containing 4′,6-diamidino-2-phenylindole (DAPI; for nuclear counterstaining). The protein gene product 9.5 (PGP 9.5; primary antibody 1:1000, Rabbit, #A01398, GenScript) was used as a neuronal marker. The S100 beta protein (S100β; primary antibody 1:500, Rabbit, #ab41548, Abcam) was used as a glial cell marker. The primary antibodies used for the detection of signaling proteins were for the particulate guanylyl cyclase G (GC-G; 1:300, Rabbit, #PGCG-701AP, FabGennix) and the cyclic nucleotide-gated channel A3 (CNGA3; 1:300, Rabbit, #LS-C14509, Lifespan Bioscience). The secondary antibody used was a Cy3-conjugated AffiniPure anti-Rabbit (Cy3; 1:200, Goat, #111-165-144, Jackson ImmunoResearch). Negative control experiments were performed by omitting primary antibodies and were also used for comparison in case of residual expression. Observations were done with LED-fluorescence microscopy (EVOS® *fl*, AMG) and acquisitions were made by confocal microscopy (SP5, Leica; LSM 710 Quasar, Zeiss) under the 40× objectives. Post-analysis and reconstructions were made with the software Imaris (Bitplane IMARIS 7.1.1).

### Calcium imaging

Calcium imaging experiments were performed on acute tissue slices of GG according to Brechbühl et al. (2008, 2013b). Animals were dissected in fresh ACSF solution at 4°C. The tips of the noses were included in blocks of 4–5% agar at a temperature <41°C and directly placed on ice for solidification. Coronal slices from 60 to 80 μm were cut and collected in ACSF at 4°C with a vibroslicer (VT1200 S, Leica). They were selected under a stereomicroscope (M165 FC, Leica) based on their general morphology, loaded in a solution of 5 μM Fura-2 acetoxymethyl ester (Fura-2AM; #0103, TEFLabs) containing 0.1% of Pluronic® F-127 (#P-6867, Invitrogen) for a minimum of 45 min in an incubator (37°C, 5% CO_2_). Slices were placed in a RC-26G bath chamber (#64-0235, Warner Instruments) and immobilized with a slice anchor. Observations were made under an inverted fluorescence microscope (Axiovert 135, Zeiss) with a 25× or 63× objective and a Cool SNAP-HQ camera. A bipolar temperature controller (SC-20/CL-100, Warner instruments) was used to control the bath temperature. The MetaFluor® system (MetaFluor, Visitron Systems) was used to monitor Fura-2AM ratio (F340/380 nm) and to acquire images (Brechbühl et al., 2011).

### Chemostimulation

The synthetic mouse alarm pheromone 2-*sec*-butyl-4,5-dihydrothiazole (SBT; Brechbühl et al., 2013b) as well as the synthetic rodent predator scents (Apfelbach et al., 2005) 2,4,5-trimethylthiazoline (TMT; #300000368, Contech), 2-propylthietane (2PT; #200000001, Contech) or the 2,3-dimetylpyrazine (Mamasuew et al., 2011a) (DMP; #W327107, Sigma) were used at 100 μM and prepared fresh before each experiment. Solution osmolarities in ACSF were between 285 and 300 Osm/L. A short perfusion of extracellular potassium (KCl; 20 mM) was used as a cellular viability test and was also used to standardize calcium responses. Intracellular calcium changes observed during the perfusion of ACSF were considered as baseline activity and increases twice larger than this baseline (corresponding to 10% of the KCl response) were considered as neuronal responses (Brechbühl et al., 2013b).

### Statistics

A minimum of 3 animals and 6 slices per conditions were used for each experimental procedure. Values are expressed as mean ± s.e.m.

## Results

### Comparative neuroanatomy of rodential grueneberg ganglia reveals structural differences

**Figure 1:**
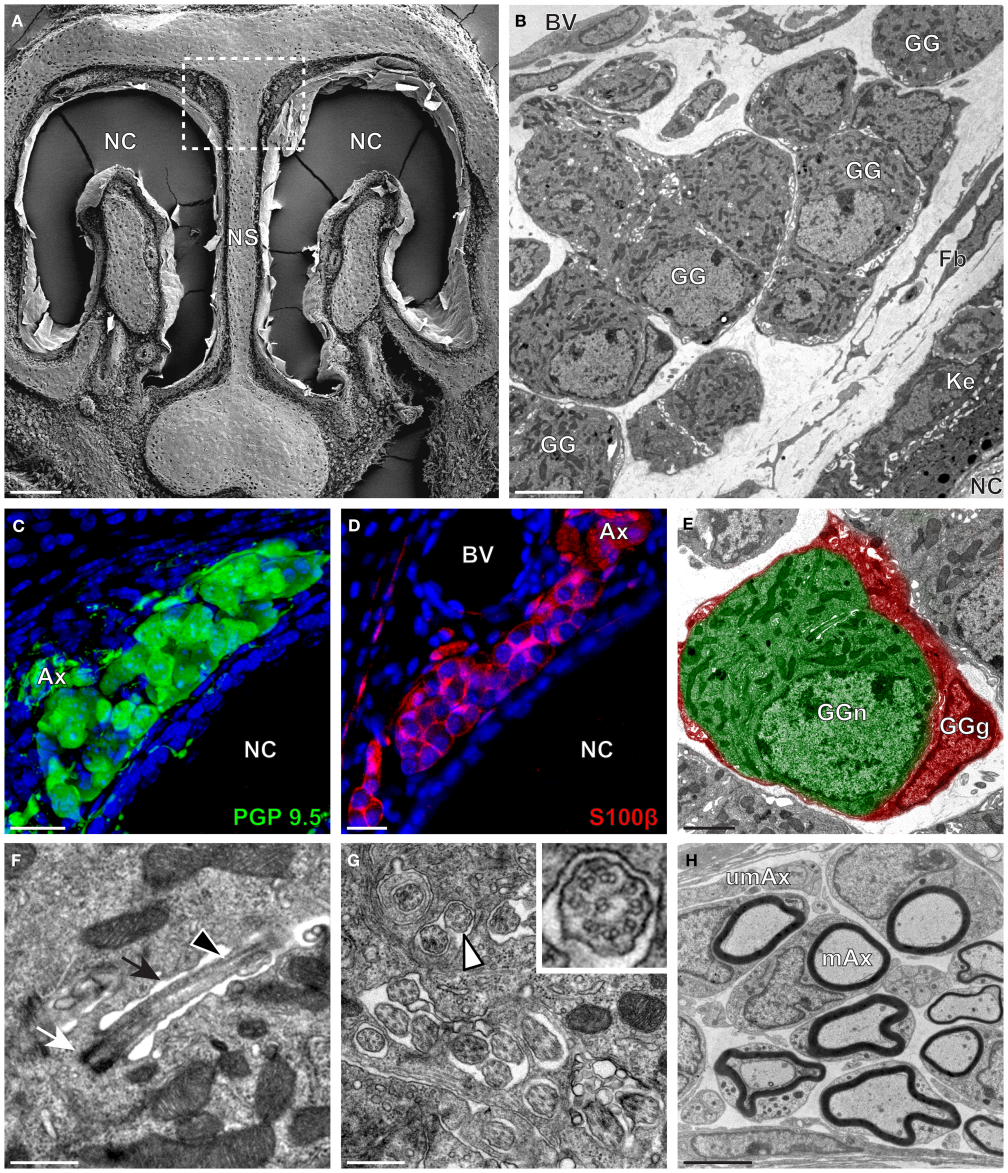
**Morphology and ultrastructural features of mouse GG. (A)** General SEM view of a coronal slice performed in a mouse (P26) GG region. The nasal septum (NS) and the nasal cavities (NC) are shown. The white dashed rectangle highlights the GG region. **(B)** TEM observation of a group of mouse (P6) GG cells. The presence of a meshwork of fibroblasts (Fb) and a keratinized epithelium (Ke) separating the GG neurons from the NC, as well as the proximity of a blood vessel (BV) can be observed. **(C,D)** Immunohistochemistry observations demonstrating the presence of both neuronal (**C**, PGP 9.5 in green, P1) and glial cells (**D**, slice view of S100β in red, P17) in the mouse GG. **(E)** High power pseudocolorized TEM view of one mouse (P6) GG neuron (GGn, in green) completely wrapped in a GG glial cell (GGg, in red). **(F,G)** Ultrastructural TEM view of mouse GGn cilia. **(F)** Sagittal observation of one mouse (P6) GGn ciliary process with its basal body (white arrow) corresponding to the beginning of the cilium as well as part of its long axoneme (black arrowhead). The GGn cell membrane (black arrow) shows the invagination of the cilium. **(G)** Coronal observation of a group of deeply invaginated mouse (P15) GGn cilia. A zoomed view of a single cilium (white arrowhead) shows the distribution of the microtubular doublets (corresponding in mouse to 8 + 1 or 9 + 0). **(H)** Coronal section of a mouse (P15) GG axonal nerve bundles (Ax). Myelinated (mAx) and unmyelinated (umAx) fibers were observed. SEM, scanning electron microscopy; TEM, transmitted electron microscopy. A minimum of 3 animals (from P1 to P31) and 6 slices per conditions were used and observed for each SEM, TEM or immunohistochemistry observations. **(C,D)** Nuclei are shown in blue (DAPI counterstain). Scale bars are 100 μm in **(A)**, 5 μm in **(B)**, 20 μm in **(C,D)**, 2 μm **(E)**, 0.5 μm in **(F,G)**, and 3 μm in **(H)**.

The neuroanatomical structure of the GG was previously described in OMP-GFP transgenic mice (Brechbühl et al., 2008; Liu et al., 2009). Here, we first observed the GG neuroanatomy of non-transgenic C57BL/6J mice (Figure 1 and Table 1) by scanning and transmitted electron microscopy (SEM and TEM, respectively) on coronal sections (Figures 1A,B). Morphologically, the GG was lacking direct contact with the nasal cavity (NC). It was situated near large BV and organized in clusters of round-ovoid cells found in fibroblast (Fb) meshworks between the nasal septum (NS) and a keratinized epithelium (Ke). The presence of the two cell populations, GG neurons (GGn) and GG glial cells (GGg), was further identified by immunohistochemistry, using specific neuronal (PGP 9.5; Figure 1C) and glial (S100β; Figure 1D) markers (Young et al., 2003) and was confirmed by high-power TEM view (Figure 1E). Cilia deeply invaginated in the neuronal soma (Figure 1F) and organized in discrete clusters with a particular microtubular doublet distribution of [proximal (8 + 1) or distal (9 + 0); (peripheral microtubular doublet + central microtubular doublet)] were observed (Figure 1G). We could differentiate, based on the high electron-density of the lamellate myelin sheaths wrapping the axonal fibers (Ax), bundles of numerous myelinated (mAx), and/or unmyelinated axons (umAx) emerging from the GG (Figure 1H). Thus, we were able to localize and characterize the presence of a GG in a non-transgenic mouse model. It displayed identical morphological features as the ones found in OMP-GFP mice (Brechbühl et al., 2008; Liu et al., 2009).

**Table 1:** **Morphological characteristics of the rodent GG**.

**Animal**	**General morphology**			**Ciliary processes per GGn**			**GG nerve**
	**GGn shape**	**Size of GGn [ μM]**	**GGg/GGn**	**Nb of cilia**	**Diameter [nm]**	**Microtubular structures**	**GG axonal bundles**
Mouse	Round-ovoid	9–14	1/(1–2)	(2–3) × (5–15)	150–250	(8 + 1); (9 + 0)	Myelinated ≅ Unmyelinated
Rat	Round	11–17	(1–2)/1	1 × (15–30)	200–300	(8 + 1); (9 + 0)	Myelinated ≅ Unmyelinated
Hamster	Flat-ovoid	12–18	1/1	(1–2) × (5–15)	150–300	(8 + 1); (9 + 0); (>9 + 0)	Myelinated > Unmyelinated
Gerbil	Ovoid	11–17	1/(2–3)	(1–3) × (10–15)	100–200	(8 + 1); (9 + 0)	Myelinated < Unmyelinated

Comparative table of the main morphological features observed in the GG of mouse, rat, hamster, and gerbil. For the general morphology, the size of each GG neuron as well as the ratio between the number of GG glial cells and the number of GG neurons are indicated (GGg/GGn). For the ciliary processes, the number of cilia per GG neuron [(n cluster) × (n cilia per cluster)], the diameter and the microtubular distribution (peripheral microtubular doublet + central microtubular doublet) are indicated. For the GG nerve, the proportion of myelinated and unmyelinated axonal bundles is listed (≅, > or <). GGn, Grueneberg ganglion neuron; GGg, Grueneberg ganglion glial cell. For each animal species and morphological criteria, a minimum of 45 GGn groups was observed.

**Figure 2:**
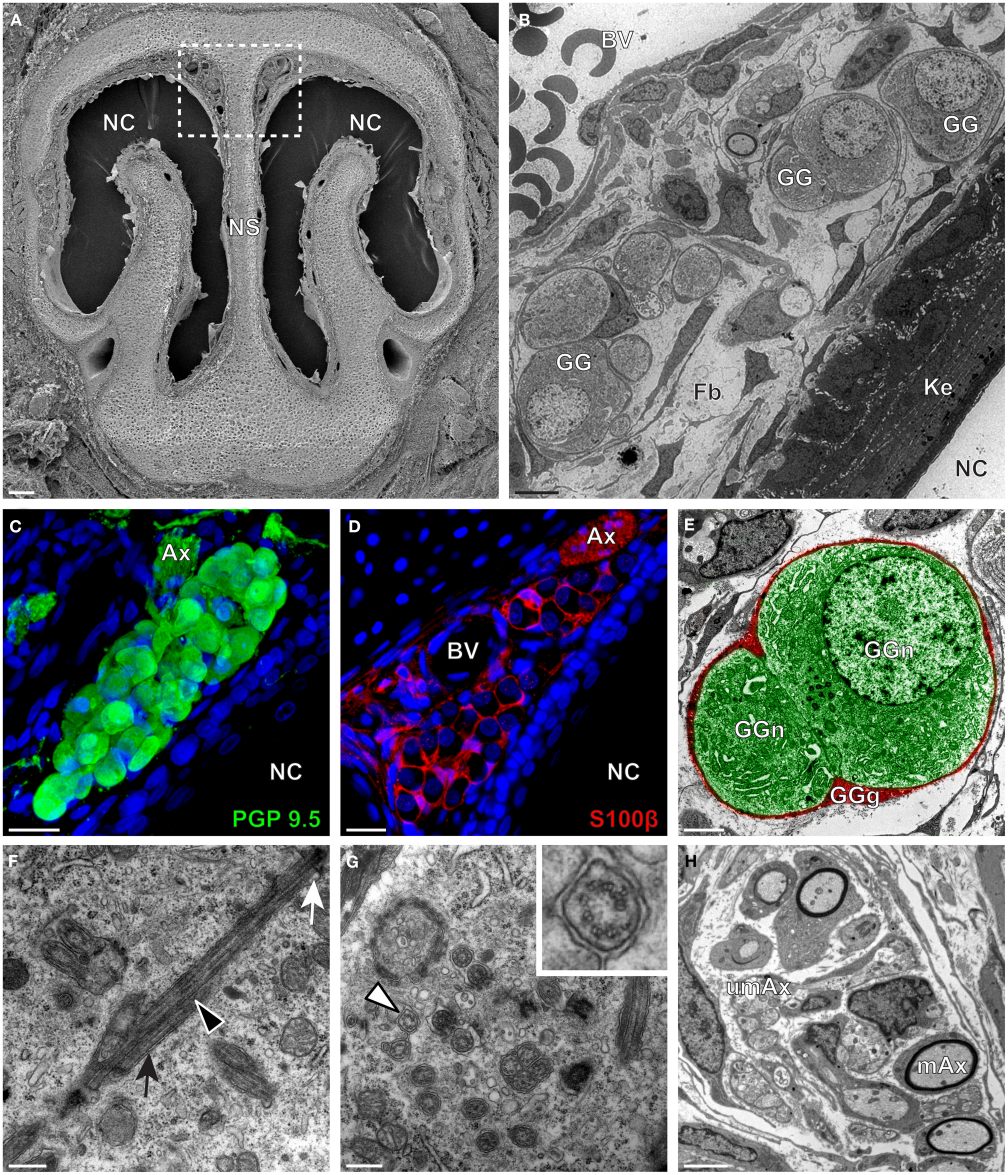
**Morphology and ultrastructural features of rat GG. (A)** General SEM view of a coronal slice performed in a rat (P7) GG region. The nasal septum (NS) and the nasal cavities (NC) are shown. The white dashed rectangle highlights the GG region. **(B)** TEM observation of a group of rat (P7) GG cells. The presence of a meshwork of fibroblasts (Fb) and a keratinized epithelium (Ke) separating the GG neurons from the NC, as well as the proximity of a blood vessel (BV) can be observed. **(C,D)** Immunohistochemistry observations demonstrating the presence of both neuronal (**C**, PGP 9.5 in green, P1) and glial cells (**D**, slice view of S100β in red, P18) in the rat GG. **(E)** High power pseudocolorized TEM view of one rat (P7) GG neuron (GGn, in green) completely wrapped in a GG glial cell (GGg, in red). **(F,G)** Ultrastructural TEM view of rat GGn cilia.** (F)** Sagittal observation of one rat (P7) GGn ciliary process with its basal body (white arrow) corresponding to the beginning of the cilium as well as part of its long axoneme (black arrowhead). The GGn cell membrane (black arrow) shows the invagination of the cilium. **(G)** Coronal observation of a group of deeply invaginated rat (P7) GGn cilia. A zoomed view of a single cilium (white arrowhead) shows the distribution of the microtubular doublets (corresponding in rat to 8 + 1 or 9 + 0). **(H)** Coronal section of a rat (P7) GG axonal nerve bundles (Ax). Myelinated (mAx) and (umAx) unmyelinated fibers were observed. SEM, scanning electron microscopy; TEM, transmitted electron microscopy. A minimum of 3 animals (from P1 to P24) and 6 slices per conditions were used and observed for each SEM, TEM, or immunohistochemistry observations. **(C,D)** Nuclei are shown in blue (DAPI counterstain). Scale bars are 100 μm in **(A)**, 5 μm in **(B)**, 20 μm in **(C,D)**, 2 μm **(E)**, 0.5 μm in **(F,G)** and 3 μm in **(H)**.

**Figure 3:**
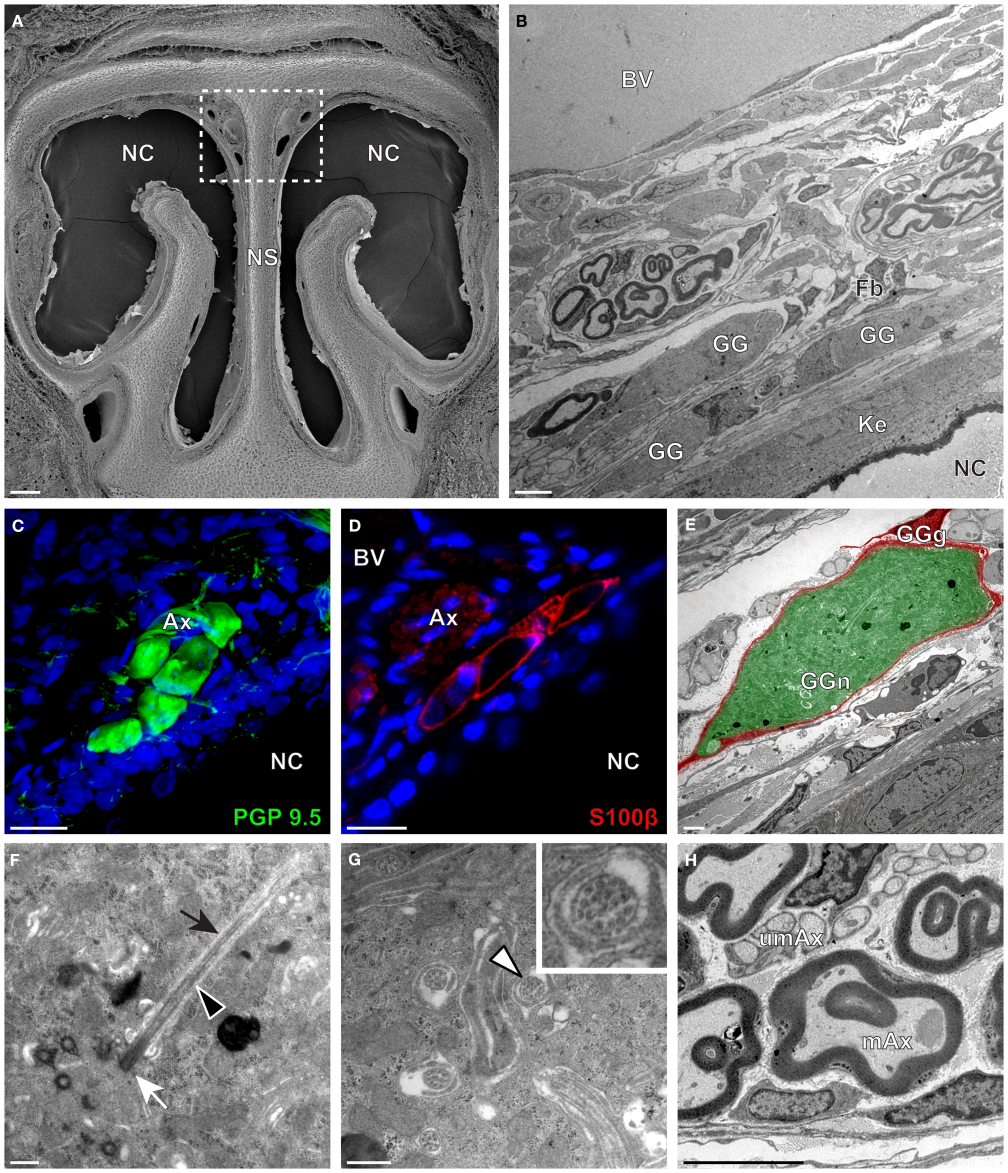
**Morphology and ultrastructural features of hamster GG. (A)** General SEM view of a coronal slice performed in a hamster (P38) GG region. The nasal septum (NS) and the nasal cavities (NC) are shown. The white dashed rectangle highlights the GG region. **(B)** TEM observation of a group of hamster (P38) GG cells. The presence of a meshwork of fibroblasts (Fb) and a keratinized epithelium (Ke) separating the GG neurons from the NC, as well as the proximity of a large blood vessel (BV) can be observed. **(C,D)** Immunohistochemistry observations demonstrating the presence of both neuronal (**C**, PGP 9.5 in green, P40) and glial cells (**D**, slice view of S100β in red, P30) in the hamster GG. **(E)** High power pseudocolorized TEM view of one hamster (P36) GG neuron (GGn, in green) completely wrapped in a GG glial cell (GGg, in red). **(F,G)** Ultrastructural TEM view of hamster GGn cilia. **(F)** Sagittal observation of one hamster (P36) GGn ciliary process with its basal body (white arrow) corresponding to the beginning of the cilium as well as its long axoneme (black arrowhead). The GGn cell membrane (black arrow) shows the invagination of the cilium. **(G)** Coronal observation of a group of deeply invaginated hamster (P34) GGn cilia. A zoomed view of a single GGn cilium (white arrowhead) shows the particular distribution of the microtubular doublets (corresponding in hamster to 8 + 1, 9 + 0, or >9 + 0). **(H)** Coronal section of hamster (P38) GG axonal nerve bundles (Ax). A predominance of (mAx) myelinated fibers was observed. SEM, scanning electron microscopy; TEM, transmitted electron microscopy. A minimum of 4 animals (from P6 to P40) and 6 slices per conditions were used and observed for each SEM, TEM, or immunohistochemistry observations. **(C,D)** Nuclei are shown in blue (DAPI counterstain). Scale bars are 100 μm in **(A)**, 5 μm in **(B)**, 20 μm in **(C,D)**, 2 μm **(E)**, 0.5 μm in **(F,G)** and 3 μm in **(H)**.

**Figure 4:**
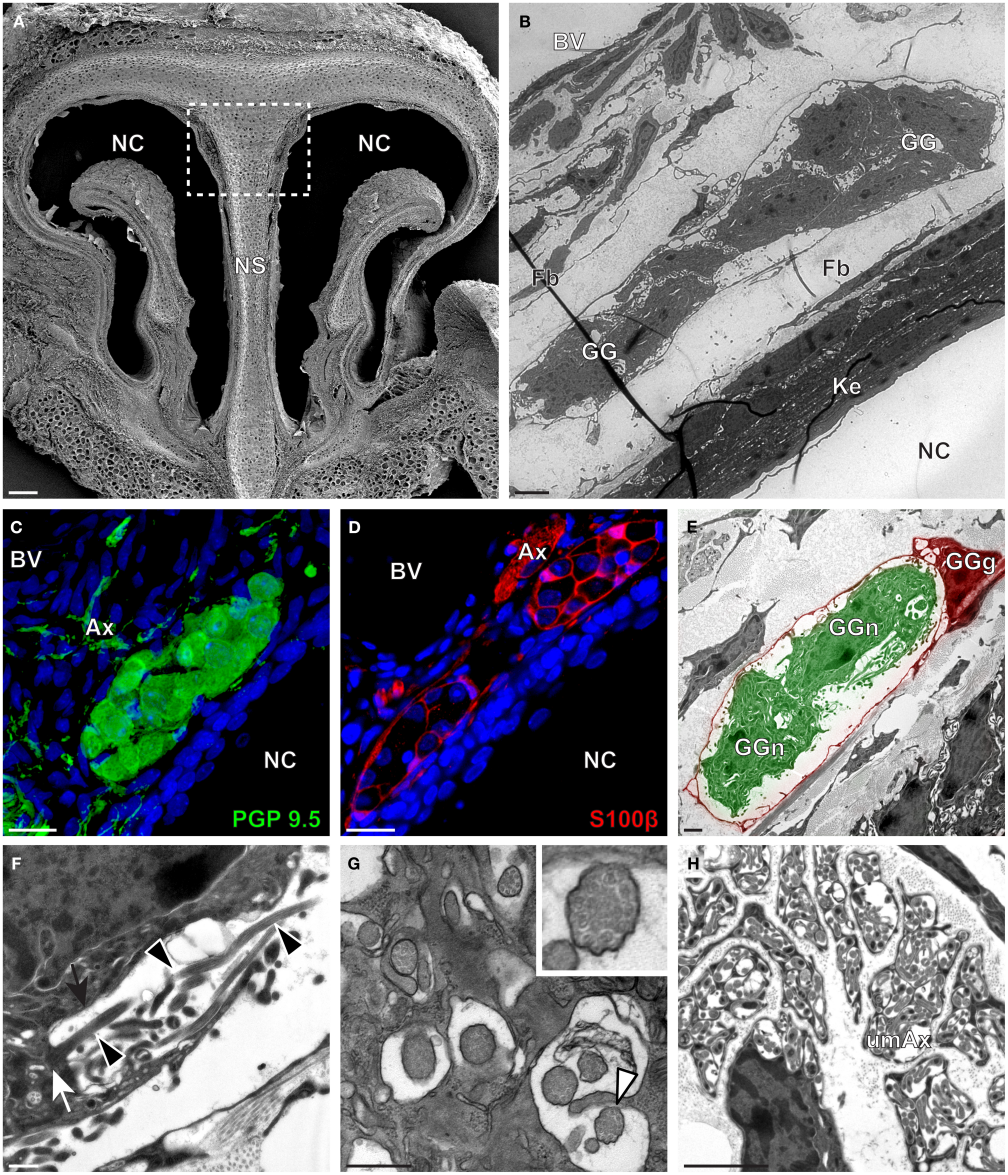
**Morphology and ultrastructural features of gerbil GG. (A)** General SEM view of a coronal slice performed in the gerbil (P10) GG region. The nasal septum (NS) and the nasal cavities (NC) are shown. The white dashed rectangle highlights the GG region. **(B)** TEM observation of a group of gerbil (P10) GG cells. The presence of a meshwork of fibroblasts (Fb) and a keratinized epithelium (Ke) separating the GG neurons from the NC, as well as the proximity of a blood vessel (BV) can be observed. **(C,D)** Immunohistochemistry observations demonstrating the presence of both neuronal (**C**, PGP 9.5 in green, P40) and glial cells (**D**, slice view of S100β in red, P12) in the gerbil GG. **(E)** High power pseudocolorized TEM view of one gerbil (P10) GG neurons (GGn, in green) wrapped partially in a GG glial cell (GGg, in red). **(F,G)** Ultrastructural TEM view of gerbil GGn cilia. **(F)** Sagittal observation of one gerbil (P10) GGn ciliary process with basal body (white arrow) corresponding to the beginning of one cilium as well long observed axoneme (black arrowhead). The GGn cell membrane (black arrow) shows the invagination of the cilium. **(G)** Coronal observation of a group of deeply invaginated gerbil (P39) GGn cilia. A zoomed view of a single cilium (white arrowhead) shows the distribution of the microtubular doublets (corresponding in gerbil to 8 + 1 or 9 + 0). **(H)** Coronal section of a gerbil (P10) GG axonal nerve bundle (Ax). A predominance of (umAx) unmyelinated fibers was observed. SEM, scanning electron microscopy; TEM, transmitted electron microscopy. A minimum of 4 animals (from P10 to P40) and 6 slices per conditions were used and observed for each SEM, TEM, or immunohistochemistry observations. **(C,D)** Nuclei are shown in blue (DAPI counterstain). Scale bars are 100 μm in **(A)**, 5 μm in **(B)**, 20 μm in **(C,D)**, 2 μm **(E)**, 0.5 μm in **(F,G)** and 3 μm in **(H)**.

We then applied these technical approaches to look for the presence of a GG in the rat (Figure 2), hamster (Figure 3), and gerbil (Figure 4). Coronal slices of the tip of the noses were obtained and the putative localization of a GG was assessed using as a template the general anatomy of the nasal cavities found in the mouse, such as the enlargement of the nasal epithelium in the dorsal region of the nasal cavities (dashed white rectangles in Figures 1A, 2A, 3A, 4A). Using these morphological criteria on the TEM preparations of different rodents, we found GG-like structures composed by cells of different shapes localized between the keratinized epithelium and the nasal septum (Figures 2B, 3B, 4B). They displayed relevant GG cellular and olfactory features as both neuronal and glial cell populations were found in these ganglion structures (Figures 2C–E, 3C–E, 4C–E) as well as the presence of numerous ciliary processes (Figures 2F,G, 3F,G, 4F,G). We thus demonstrate here neuroanatomically the presence of a GG in the rat, hamster and gerbil.

Interestingly, important species-dependent differences were observed in the structural morphologies of these GG (Table 1). By comparison, large grapes of GG cells were found in the mouse, rat, and gerbil while in hamster, only isolated or small groups of GG cells were sporadically distributed below the keratinized epithelium (Figures 3B–D and Table 1). Using a combination of electron microscopy and immunohistochemistry approaches, we also found that the glial-neuronal ratio (GGg/GGn) was not conserved between rodent species. In gerbils, this difference was particularly important as more than one GG neuron was often wrapped by a single glial cell (Figures 4D,E and Table 1). Interestingly, interspecies differences were also found at the ultrastructural level. For example, the long neuronal ciliary processes were different in their distribution and in their number. Indeed, in rats, only a single discrete cluster of ciliary processes was observed (Figures 2F,G and Table 1) whereas in other rodents more than one cluster could be found. The distribution of intraciliary microtubules also diverged between rodent species. While their typical doublet configuration of (8 + 1) at the proximal part of the cilium and of (9 + 0) along the distal axonem (Brechbühl et al., 2008) was always observed, an additional distal configuration of (>9 + 0) was often found in hamsters (Figure 3G and Table 1). At the nerve level, the GG axonal bundles were also divergent (Figures 1H, 2H, 3H, 4H and Table 1). Indeed, numerous myelinated and unmyelinated axons were found in mice, rats, and hamsters, while a predominance of lightly myelinated and/or unmyelinated fibers were observed in gerbils (Figure 4H and Table 1). In summary, we found a GG in each investigated rodent but divergent structural features were observed that might point to a species-dependent function of this olfactory subsystem.

### Rodential grueneberg ganglion neurons display a differential expression of cGMP-related signaling proteins

**Figure 5:**
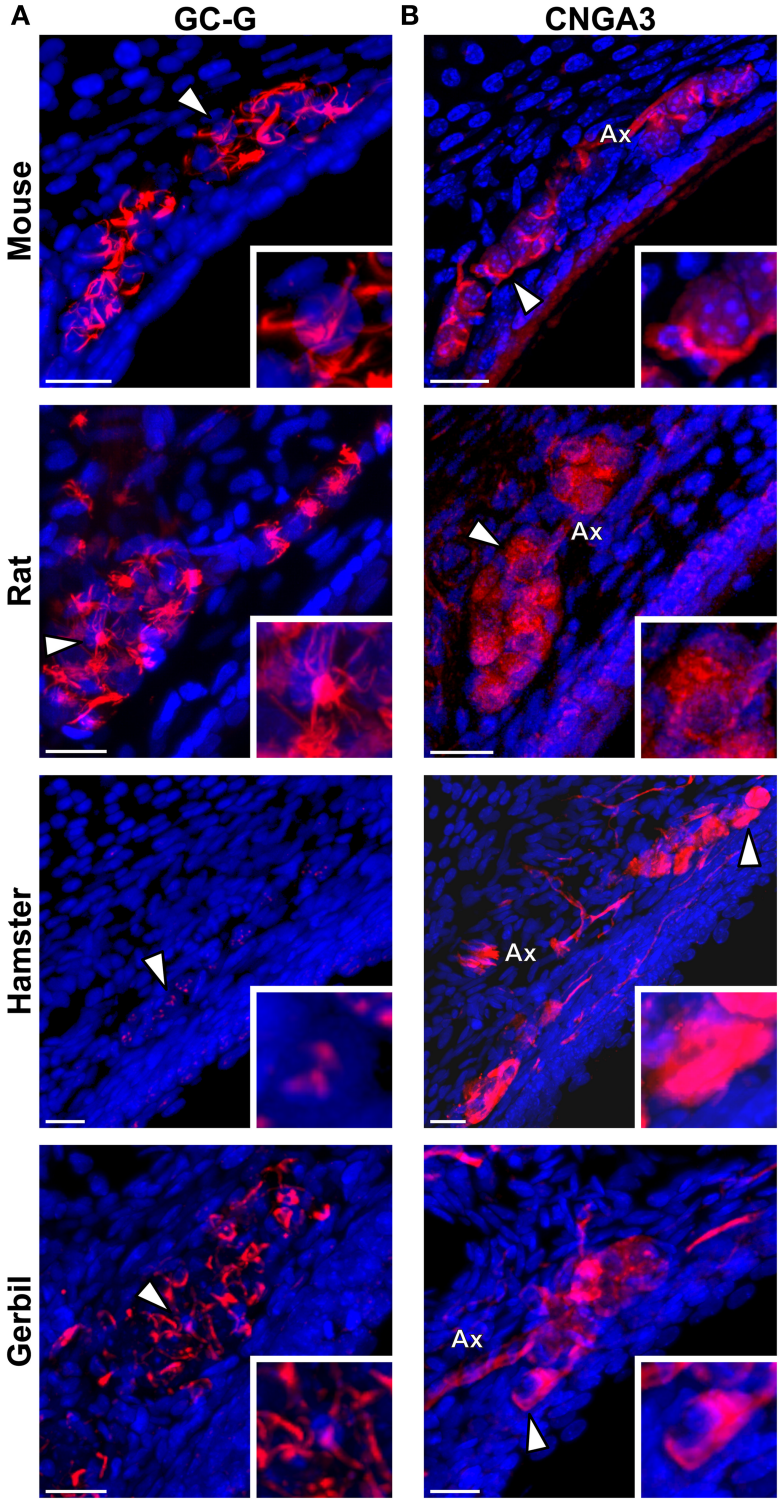
**Species-dependent expression of cGMP-associated signaling proteins in the rodential Grueneberg ganglia**. Immunohistochemical investigations performed for GC-G and CNGA3 proteins in GG coronal slices from mouse, rat, hamster, and gerbil. **(A)** GC-G was found in mouse, rat, and gerbil GGn cilia. Residual expression was observed in hamster. **(B)** CNGA3 was found principally in GGn cilia and axon (Ax) with partial somatic expression in mouse, while in the other rodents widespread expressions were detected. **(A,B)** White arrowheads indicate the neuron illustrated in the high power view inset. A minimum of 3 animals (mouse: from P1 to P26; rat: from P3 to P29; hamster: from P2 to P16; gerbil: from P1 to P30) and 6 slices were used for each staining and species. Nuclei are shown in blue (DAPI counterstain). Scale bars, 20 μm.

We then verified if the known signaling proteins expressed in mouse GG neurons were expressed in the GG of other rodents. We performed immunohistochemical investigations on coronal GG slices, focusing on cGMP-related signaling proteins reported to be implicated in mouse GG sensory transductions (Mamasuew et al., 2010, 2011b; Hanke et al., 2013). Thus, the expression of the particulate guanylyl cyclase G (GC-G), a potential transmembrane receptor and of the cyclic nucleotide-gated channel A3 (CNGA3) a non-selective cation channel, both expressed in the mouse GG (Fleischer et al., 2009; Liu et al., 2009; Brechbühl et al., 2013a), were examined (Figure 5). We found that these signaling proteins were expressed differently in the investigated rodents (Table 2). GC-G was clearly found in the GG ciliary processes of the mouse, rat and gerbil but only residual expression was found in hamster (Figure 5A). The expression of CNGA3 varied also (Figure 5B). In the mouse GG, CNGA3 was found to be principally expressed in cilia and axons with some somatic localization (Brechbühl et al., 2013a), while in the other rodents widespread distribution was detected. Thus, both the expression and the localization of these GG signaling proteins were found to be species-dependent, which may reflect neurosensory disparities.

**Table 2:** **Physiological properties of the rodent GG**.

**Animal**	**Signaling proteins**				**Chemosensitivity**								**Thermosensitivity**	
	**GC-G**		**CNGA3**		**SBT**		**TMT**		**DMP**		**2PT**		**25–10°C**	
Mouse	++	Cilia	++	Cilia, axon	+	21/23	++	33/33	+	23/24	++	17/17	+	11/14
Rat	++	Cilia	+	Widespread	(+)	3/12	++	27/29	+	12/16	+	34/41	+	10/14
Hamster	(+)	n.d	+	Widespread	+	9/9	+	12/16	++	13/13	+	6/9	(+)	4/11
Gerbil	++	Cilia	+	Widespread	−	2/14	(+)	21/57	(+)	28/32	−	2/14	++	11/14

Comparative table of the physiological criteria observed in the GG of mouse, rat, hamster, and gerbil. For the signaling proteins, the expressions (++, highly expressed; +, expressed; (+), residual expression) as well as the main identified subcellular-localization [cilia, axon, widespread, or n.d (not defined)] of both GC-G and CNGA3 are mentioned. For the chemo- and the thermo-sensitivity, the mean response intensity (++, highly sensitive; +, sensitive; (+), poorly sensitive; −, not sensitive) and the proportion of responding cells (n responding neurons/n tested and viable neurons) for SBT, TMT, DMP, 2PT at 100 μM and to cold temperature variation (from 25 to 10°C) are indicated. GC-G, particulate guanylyl cyclase G; CNGA3, cyclic nucleotide-gated channel A3.

### Chemo- and thermo-evoked sensory activities of the grueneberg ganglion are species-dependent

**Figure 6:**
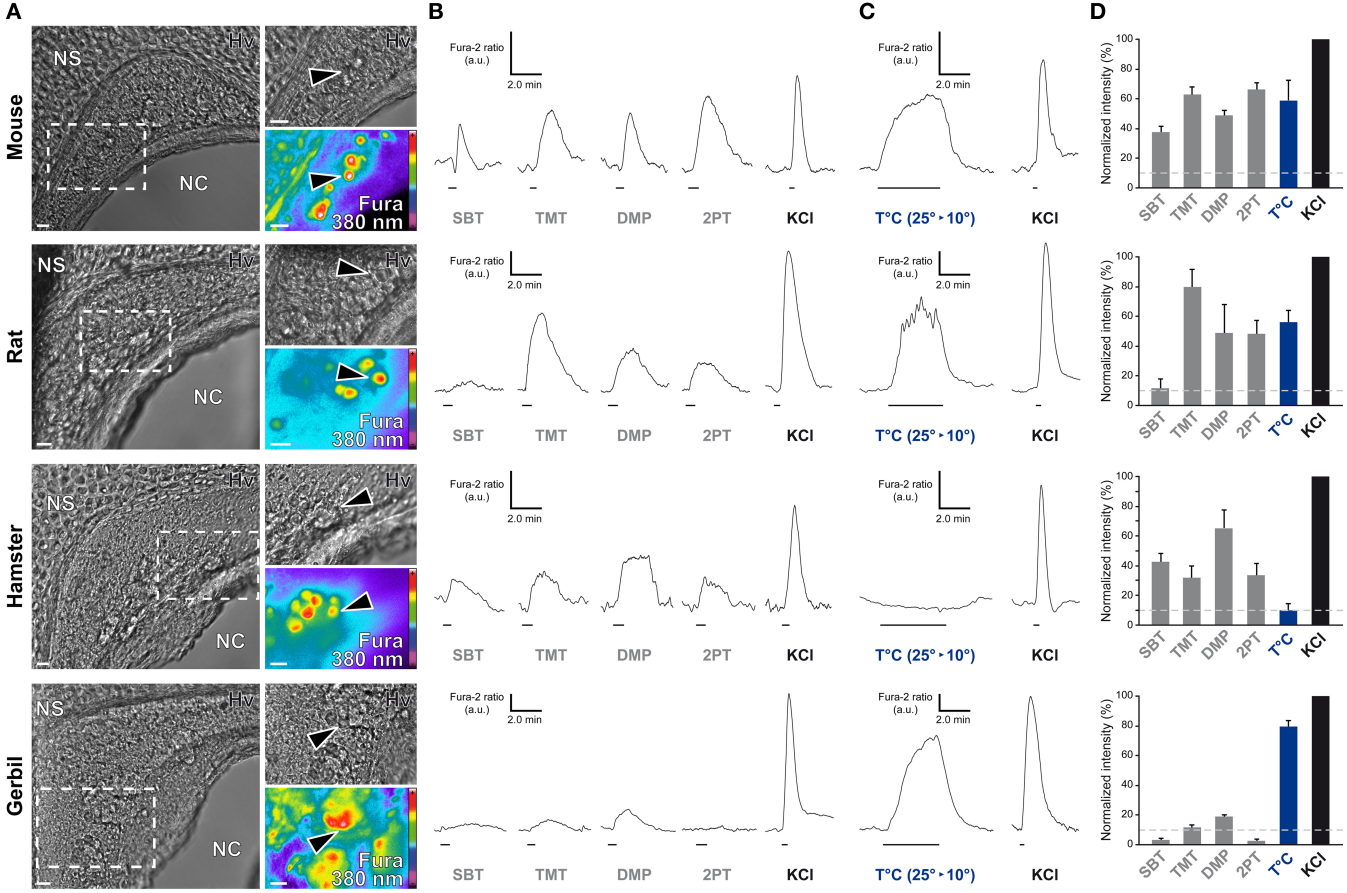
**Species-dependent characteristics of sensory responses in rodential Grueneberg ganglion neurons. (A)** GG coronal slices from mouse, rat, hamster, and gerbil where GG neurons can be observed under Hoffman modulation view (Hv) between the nasal cavity (NC) and the nasal septum (NS). White dashed rectangles indicate the high power view regions used for recording neuronal activities. The fluorescence of Fura-2AM into GG neurons observed at 380 nm in a color encoded map for unbound Fura is illustrated here. Black arrowheads indicated GG neurons used for the traces found in **(B)** and/or in **(C)**. **(B)** Representative chemo-evoked responses observed at RT after the successive perfusion of SBT, TMT, DMP, or 2PT (100 μM) on the same GG neurons. **(C)** Representative thermo-evoked responses observed during a cold stimulation (T°C; decrease from 25 to 10°C). Here calcium increases are shown for mouse, rat and gerbil. In hamster, the illustrated chemo-responding neuron (in **B**) did not respond to the thermal stimulation. **(D)** Histograms summarizing Fura-2 ratio peak calcium responses intensity to chemo- and thermo-stimulations (respectively in gray and blue bars) normalized to KCl responses (20 mM; in black bars). 10% of KCl activity (gray dashed lines) was used as a limit for considering a neuronal response. Values are expressed as mean ± s.e.m. A minimum of 3 animals (mouse: from P0 to P6; rat: from P1 to P4; hamster: from P2 to P4; gerbil: from P0 to P3) and 6 slices were used for each species. Scale bars, 20 μm in **(A)**. Fluorescence intensity Fura-2 ratio = F340/F380 is indicated by arbitrary units (a.u.) in **(B)** and **(C)**. Perfusion times for chemo- and thermo-stimulations are indicated by horizontal bars in **(B)** and **(C)**.

In the mouse, the GG is implicated both in chemo- and thermo-detection (Fleischer and Breer, 2010). Indeed, structurally similar thiazolic- and pyrazine-derived compounds (Mamasuew et al., 2011a; Brechbühl et al., 2013b) found for example in predator scents or emitted by stressed mice (Apfelbach et al., 2005; Brechbühl et al., 2013b) initiated, in the majority of GG neurons, strong reversible and physiological responses. Until now, the most potent molecules reported to generate neuronal activities are 2-*sec*-butyl-4,5-dihydrothiazole (SBT; a mouse alarm pheromone), 2,4,5-trimethylthiazoline (TMT; a red fox scent), 2-propylthietane (2PT; a stoat scent) (Brechbühl et al., 2013b), or 2,3-dimetylpyrazine [DMP (Mamasuew et al., 2011a); a molecule closely related to substances present in the urine of predators]. Temperature variations have also been reported to induce neuronal activities in the mouse GG (Mamasuew et al., 2008; Schmid et al., 2010; Stebe et al., 2013). For example, a cold environmental temperature initiated calcium elevations in most mouse GGn (Schmid et al., 2010; Brechbühl et al., 2013a). Here, we first established acute tissue slice preparations from GG regions of the rat, hamster, and gerbil to perform calcium imaging experiments (Figure 6A). The different rodent GG were identified by their specific morphologies and their Fura-2AM loading. We then compared chemo-evoked activities across species by perfusing GGn with SBT, TMT, DMP, or 2PT [100 μM; (Brechbühl et al., 2013b)] in a continuous oxycarbonated ACSF perfusion (Figure 6B). Thermo-evoked neuronal activities induced by a transient decrease in temperature from 25 to 10°C were also compared (Figure 6C). Neuronal calcium responses were measured and their intensities were normalized to a brief stimulation with KCl (Figure 6D). We found that GGn responded differently across rodents (Figure 6D and Table 2). As previously reported, in the mouse, strong neuronal responses were evoked by SBT, TMT, DMP, 2PT and by temperature stimuli in the majority of GGn (91, 100, 96, 100, and 79% of GGn, respectively; *n* total of viable recorded neurons = 81). In the rat, similar activities were recorded (25, 93, 75, 83, and 71%, respectively; *n* = 58), interestingly a reduction in the proportion of responsive neurons and in the calcium signals were observed for the mouse alarm pheromone SBT. In the hamster, chemo-evoked responses were also observed but a drastic decrease in thermo-evoked responses was found both for the number of temperature responding GGn and the mean normalized intensity of the responses (100, 75, 100, 67, and 36%, respectively; *n* = 20). On the contrary, in the gerbil chemo-evoked responses were considerably reduced but the thermo-evoked responses were present (14, 37, 88, 14, and 79%, respectively; *n* = 64). Thus, we show here that the acute tissue slice preparation first developed in the mouse could be applied in the rat, hamster and gerbil. Furthermore, this physiological model allowed the observation that species-dependent sensory activities occur in the different rodent Grueneberg ganglia.

## Discussion

Rodents have varied geographical distributions and live in different ecological niches. Across evolution, their senses have adapted to their habitats and they have therefore developed a series of strategies and behaviors to survive in their environments (Kavaliers and Choleris, 2001). Detection of danger signals such as chemical warnings emitted by their conspecifics (alarm pheromones) or predator-produced cues (kairomones) takes place in the different olfactory subsystems present in their nasal cavities, which then convey multiple sensory informations (Ma, 2010). To increase their survival strategies and fitness, adaptation of the air-breathing pathway in nasal cavities as well as the general nose morphology itself have facilitated this specific olfactory detection (Negus, 1954; Harkema, 1991; Harkema et al., 2006; Rae et al., 2006). This environmental adaptation is especially significant for the most rostral part of the rodent wet-snout, the so-called rhinarium (Hill, 1948; Ade, 1999), which is implicated both in tactile and active olfactory sensing (sniffing behavior) (Wachowiak, 2011; Haidarliu et al., 2012, 2013) and is directly dependent of the environmental context such as temperature variations (Ade, 1999; Ince et al., 2012; Brechbühl et al., 2013a; Cilulko et al., 2013).

We here focused on the GG, an olfactory subsystem devoted to the detection of environmental stress (Brechbühl et al., 2008, 2013b), which is located in the rhinarium region (Haidarliu et al., 2012, 2013; Brechbühl et al., 2013a). We speculated that, due to this specific rostral localization and its temperature sensitivity, it has been influenced by the environmental pressure and has modified its structural morphology and physiological sensory abilities across species by an evolutionary process, conferring a putative ethological function to the GG. We therefore looked at the comparative anatomy and physiology of this olfactory subsystem in four different rodent models that live in distinctive ecological niches. We have first confirmed here by histological investigations the presence of a GG in the mouse, rat, and hamster (Grüneberg, 1973) and demonstrate its existence in the gerbil. We found that GG structural morphologies indeed vary; in particular in the hamster where the gross morphology of the GG was different compared to the other rodents. The hamster GGn have a flat-ovoid shape and are distributed sporadically in small groups of cells across the nasal epithelium at the tip of their noses. This morphological anatomical specificity might be a neuroanatomical adaptation, probably due to the large and flat hamster's nose that might limit the ganglion extension observed in the other investigated rodents. By these anatomical criteria, the hamster's rhinarium is closer to the center of the animal's body and thus should be less affected by external temperature variations than those of the rat and mouse (Sokoloff and Blumberg, 2002; Brechbühl et al., 2013a), a hypothesis reinforced here by the low-sensitivity of the hamster's GGn to temperature variations as observed by calcium imaging experiments. On the other hand, the high temperature sensitivity displayed by the gerbil GGn seems to be correlated with the environmental thermal stress of its desert habitat (extreme variations in day/night temperatures) that strongly influences the behaviors and the temperature of the body extremities (Thiessen, 1988; Klir et al., 1990). Furthermore, the chemosensitivity of GGn as observed by our calcium imaging experiments seems to be altered in gerbils compared to the other investigated rodents. This physiological particularity could similarly be linked to the precise morphology of the gerbil GG. Indeed in the gerbil, the GGg/GGn ratio is the lowest observed. As in our acute tissue-slice preparation the glial wrapping context of the neurons is preserved, we can therefore speculate that glial cells might thus interact with the chemosensitivity of the GGn. A situation also found in the olfactory system of other organisms such as the amphid neurons of *C. elegans* in which the genetic deletion of the wrapping glial cells drastically alter the chemo-sensory function of the neurons (Bacaj et al., 2008). The apparent low responsiveness of the gerbils GGn could also be explained by a different repertoire of expressed receptors and affiliated chemical ligands, which could also be further investigated. Detailed morphological investigations, based on serial sections through the rodents GG and/or olfactory bulb, would also determine the longitudinal organization of the GG (number of GGn per individual) as well as the central projections (necklace glomeruli) in the different rodents and thus potentially be associated with the physiological differences observed.

In addition to the morphological properties, the species-dependent expression of cGMP-related signaling proteins in the GGn of the different rodents tested could also be correlated with the physiological responses observed. We cannot rule out that our immunostaining experiments might have been influenced by the antibody species-affinity. Nonetheless, we took advantage for example that the epitope of the GC-G antibody is at the C-terminal region of the protein, which shares approximately 95% identity between the mouse (gi: 124487301) and rat (gi: 404351639) or between the mouse and the predicted hamster GC-G sequence (gi: 524918444). We found, for example, that the expression of GC-G seems to be required for thermo- rather than for chemo-sensitivity. Indeed, only residual expression of the GC-G protein was found in the less temperature-sensitive hamster GG. Putative expression of other GC-members (Liu et al., 2009) could substitute for this GC chemo-related signaling function and explain the chemo-sensitivity found in hamster GGn. Other signaling proteins expressed in mice GGn, such as the V2r83 (member of the V2R class of olfactory receptors) (Fleischer et al., 2006b) or the cGMP-dependent protein kinase of type II (cGKII) (Liu et al., 2009) were not investigated here. The signaling protein expression seem to be species-dependent, it could therefore be interesting to verify the expression of these proteins in the other rodent species and to further investigate their potential implication in the unknown transduction signaling cascades and physiological responses of GG neurons.

The mouse GG was shown to be involved in the recognition of volatile danger cues such as alarm pheromones and other structurally-related molecules involuntary released by rodents terrestrial predators (Brechbühl et al., 2008, 2013b). In mammals, alarm pheromones are complex and composed by more than one molecule (Kiyokawa et al., 2004). They have not been chemically identified in all rodent species. 2-heptanone has been proposed as a putative rat alarm pheromone (Gutierrez-Garcia et al., 2007). This pheromonal compound did not induce responses in the mouse GG (Brechbühl et al., 2008, 2013b; Mamasuew et al., 2011a) and in the GG of the other species tested (data not shown). This chemodetection seems to be mediated in the rat by another olfactory subsystem, the vomeronasal organ (VNO) (Kiyokawa et al., 2013) pointing out to the complexity of the detection of chemical danger cues. Interestingly, the identified mouse alarm pheromone SBT (Brechbühl et al., 2013b) was perfused on rodents GGn and its activation seems to be specific to the mouse and hamster confirming, at the molecular level, the hypothesis that alarm pheromones could be shared warning signals (Apfelbach et al., 2005).

For the other danger signaling chemicals, the predator cues, the GG-evoked responses observed were also different across species and were correlated with the predator-prey context. For example, the fox is a natural predator of rats. It is known to initiate in the rat important stereotypical fear-related behaviors and an increase of plasmatic stress-related hormones (Apfelbach et al., 2005; Kobayakawa et al., 2007; Ferrero et al., 2011). In our study, we used TMT as a fox scent in perfusion and it generated the highest observed calcium responses in rats GG, thus reinforcing the concept of a potential ethological function for this olfactory subsystem as well as the ecological notion of the predator-prey detection ability due to the habitat shift or learning process (Apfelbach et al., 2005; Kass et al., 2013). Based on this, it could be interesting to examine the presence of a GG and its sensitivities to kairomones in other species including mammals from other ecological niches that did not develop innate fear behaviors in the presence of terrestrial predator scents such as bats (Driessens and Siemers, 2010).

Taken together, the ability of the GG to detect a large array of danger cues with various efficacies in the same neuron and across rodents, raised the question of the potential expression of multiple olfactory receptors and/or multiple signaling pathways at the GG organ level (Fleischer et al., 2006b, 2007; Pyrski et al., 2007; Schmid et al., 2010; Liu et al., 2012; Stebe et al., 2013) but also at the single GG neuronal level (Brechbühl et al., 2013a). As demonstrated for another olfactory subsystem, the VNO (Salazar and Quinteiro, 2009), the presence of a sensory organ that shares gross morphological similarities may be taken into account with limitation to extrapolate physiological notions between species. Indeed, we show here that the GG has conserved a general similar morphology across the investigated rodent species nevertheless its sensory characteristics are strikingly species-dependent pointing to a probable evolutionary benefit for the survival of the species.

## Author contributions

Julien Brechbühl and Marie-Christine Broillet designed research; Julien Brechbühl, Magali Klaey, Fabian Moine, Esther Bovay, Nicolas Hurni, Monique Nenniger-Tosato performed research; Julien Brechbühl, Magali Klaey, and Marie-Christine Broillet analyzed data; and Julien Brechbühl and Marie-Christine Broillet wrote the paper.

### Conflict of interest statement

The authors declare that the research was conducted in the absence of any commercial or financial relationships that could be construed as a potential conflict of interest.
